# Gut Microbiota-specific Profile Prior to Surgery for Predicting Type 2 Diabetes Remission in Patients Undergoing Sleeve Gastrectomy

**DOI:** 10.1007/s11695-026-08612-6

**Published:** 2026-03-26

**Authors:** José Ignacio Martínez-Montoro, Raquel Sancho-Marín, Lourdes Garrido-Sánchez, Luis Ocaña-Wilhelmi, Rocío Soler-Humanes, Nerea Ruiz-Campos, María José García-López, Francisco J Tinahones, Carolina Gutiérrez-Repiso

**Affiliations:** 1 Department of Endocrinology and Nutrition, Virgen de la Victoria University Hospital, Málaga, Spain; 2 Instituto de Investigación Biomédica de Málaga y Plataforma en Nanomedicina–IBIMA Plataforma Bionand, Málaga, Spain; 3 Centro de Investigación Biomédica en Red-Fisiopatología de la Obesidad y Nutrición (CIBEROBN), Madrid, Spain; 4 Department of General and Digestive Surgery, Virgen de la Victoria University Hospital, Málaga, Spain; 5 Faculty of Medicine, University of Málaga, Málaga, Spain

**Keywords:** Type 2 diabetes, sleeve gastrectomy, gut microbiota, diabetes remission

## Abstract

**Objective:**

The predictive role of baseline gut microbiota in type 2 diabetes (T2D) remission after bariatric surgery remains unexplored. This study aimed to identify specific gut microbiota profiles prior to surgery associated with T2D remission following sleeve gastrectomy (SG).

**Methods:**

This was an observational study including participants with a body mass index (BMI) **≥** 40 kg/m^2^ and T2D who underwent SG, and had preoperative stool samples available. Gut microbiota was analayzed by 16 S rRNA sequencing. Participants were classified into remission and non-remission groups based on T2D status one year after SG.

**Results:**

A total of forty-six participants were included. After adjusting for baseline confounders (i.e., age, HbA1c levels, T2D duration, and insulin treatment), preoperative gut microbiota diversity showed no statistically significant differences between groups, except for Pielou’s evenness index, which was significantly higher in the non-remission group (*p* = 0.01). ANCOM-BC2 analysis identified an enrichment in *Fusicatenibacter*, *Holdemanella* and *Senegalimassilia* in the non-remission group, whereas *Eggerthella*,* Flavonifractor*,* Ruminococcaceae g__Incertae Sedis* and *Ruminococcus gnavus group* were enriched in the remission group. Furthermore, insulin treatment and the gut microbial taxa *Ruminococcaceae g__Incertae Sedis*, *Fusicatenibacter*, and *Eggerthella* emerged as potential predictors of T2D remission. Functional analysis using PICRUSt2 revealed increased carbohydrate metabolism pathways in the remission group.

**Conclusions:**

Baseline gut microbiota composition may serve as an independent predictor of T2D remission in patients undergoing SG, and could become a potentially relevant biomarker, complementing other existing clinical predictors.

**Graphical Abstract:**

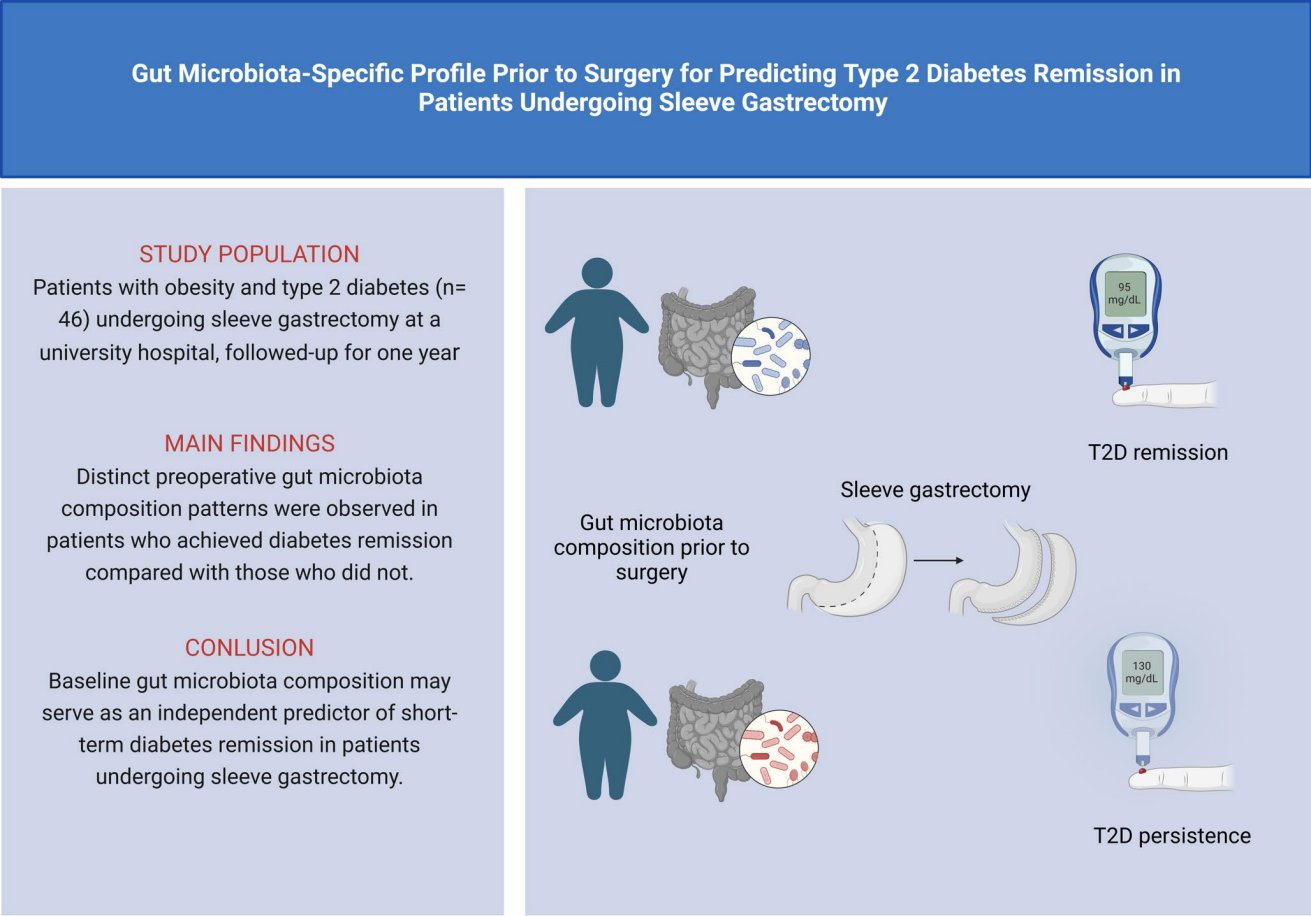

**Supplementary Information:**

The online version contains supplementary material available at 10.1007/s11695-026-08612-6.

## Key points

The predictive role of baseline gut microbiota in type 2 diabetes (T2D) remission after sleeve gastrectomy (SG) remains unexplored.

We show that distinct preoperative gut microbiota composition patterns can be observed in patients who achieve T2D remission compared with those who do not.

Baseline gut microbiota composition may serve as a biomarker of T2D remission in patients undergoing SG.

**Supplementary Information:**

The online version contains supplementary material available at 10.1007/s11695-026-08612-6.

## Introduction

Type 2 diabetes (T2D) is a chronic disease characterized by insulin resistance followed by a progressive decline in pancreatic β cell function, with an increasing prevalence worldwide [1, 2]. Both genetic and environmental factors are involved in the pathophysiology of T2D, with the rising obesity pandemic as key cause for the development of the disease [1, 2]. Notably, in recent decades, the gut microbiome (defined as the gut microbiota -i.e., the community of microorganisms that inhabits the gastrointestinal tract- together with its collective genomes, and metabolites) has also been associated with T2D [3, 4].

Although different therapeutic strategies are available for the management of T2D, including diet, physical activity, and glucose-lowering medications, bariatric surgery (BS), has been demonstrated to be the most effective treatment for this disease to date, being associated with higher rates of T2D remission following the procedure [5, 6]. Importantly, among the factors leading to T2D remission following BS, some associations with the gut microbiota have been described. In this regard, previous studies have suggested differential changes in gut microbiota after BS according to T2D remission. Thus, specific gut microbiota signatures associated with enhanced glucagon like peptide 1 and 2 (GLP-1 and GLP-2) secretion have been identified in patients with T2D with improved metabolic control after Roux-en-Y gastric bypass (RYGB) [7]. On the other hand, the absence of T2D resolution after RYGB may be linked to an increase in specific bacterial species, such as *Phocaeicola dorei*,* Bacteroides fragilis*, and *Bacteroides caecimuris*, following surgery [8]. It should also be noted that differential modifications in gut microbiota composition after BS according to T2D persistence or remission may occur between different surgical techniques (i.e., RYGB and SG) [9]. In fact, several changes in gut microbiota composition and related metabolites after SG have been associated with T2D remission [10]. Additionally, prior studies have examined baseline gut microbiota or post-BS changes and their predictive role in weight loss outcomes [11, 12]. However, whereas different factors prior to surgery, such as age, diabetes duration, glycated hemoglobin (HbA1c) levels or insulin therapy, have been demonstrated to influence T2D persistence/remission after BS [13–15], the role of baseline gut microbiota in T2D resolution after BS has remained poorly explored. Previously, Davies et al. found a pre-surgery increase in the abundance of *Eubacteriaceae* and *Alistipes putredinis* in patients who achieved T2D remission one year after BS (22 participants undergoing RYGB and 22 undergoing SG) [16]. However, this study did not analyze these surgical techniques separately, or account adjusting for potential, well-known clinical baseline confounders associated with T2D remission. Another study involving 14 women that underwent RYGB identified a relationship between baseline gut microbiota composition and T2D remission one year post-surgery, and patients who achieved T2D remission had significantly lower abundances of *Asaccharobacter* and *Atopobium*, and higher abundances of *Gemella*, *Coprococcus*, and *Desulfovibri*o [17]. Nevertheless, to our knowledge, no further studies have evaluated the impact of baseline gut microbiota composition on T2D remission after BS, particularly in patients undergoing SG, the most commonly performed BS technique worldwide.

In this study, we aimed to identify specific gut microbiota profiles prior to surgery associated with T2D remission or persistence one year after SG.

## Methods

### Participants

This observational prospective study was conducted from 2022 to 2024 and included participants with severe obesity undergoing BS at Virgen de la Victoria University Hospital (Málaga, Spain).

The inclusion criteria comprised patients with a body mass index -BMI- **≥** 40 kg/m^2^ and a previous diagnosis of T2D; laparoscopic SG as surgical technique, available preoperative stool sample and completed scheduled follow-up visits. The flowchart of the study participants is shown in Supplementary Fig. 1. All study participants followed a very low-calorie diet (VLCD) (600 to 800 kcal) for 15 days before surgery using Optisource (Nestlé Health Care Nutrition) supplemented with Proteplus NM protein (1 g/kg of ideal body weight, based on a BMI of 25 kg/m^2^). The stool sample was obtained prior to the VLCD.

The exclusion criteria were prior cardiovascular disease (myocardial infarction, unstable angina, stroke, peripheral artery disease or coronary revascularization), acute inflammatory conditions (including active infection, fever, or active autoimmune disease), infectious disease, history of bowel resection, prior bariatric procedures, or surgical technique other than SG. To minimize potential confounding factors affecting the gut microbiota, the use of antibiotics, probiotic, or prebiotic agents in the previous 3 months were reasons for exclusion.

Participants were classified into two groups according to T2D remission one year after SG. T2D remission was defined according to international criteria as HbA1c < 6.5% for at last three months off glucose-lowering medications [18].

This study was reviewed and approved by the Ethics Research Committee of Málaga, and was conducted according to the Declaration of Helsinki. All participants gave their written informed consent to participate in this study.

### Clinical, Anthropometric and Laboratory Measurements

Prior to the surgical intervention and again at the one-year follow-up, all participants underwent a standardized assessment of anthropometric parameters. BMI was determined by dividing weight in kilograms by the square of height in meters. Blood samples were obtained from fasting patients (after 10–12 h of fasting) at both time points. Serum was separated via centrifugation and stored at − 80 °C until further analysis. Fecal samples were collected in stool specimen collectors and immediately stored at -80 °C until processing.

Serum total cholesterol, high-density lipoprotein cholesterol (HDL-c), triglycerides, and glucose were measured by the Advia Chemistry XPT autoanalyzer (Siemens Healthcare Diagnostics). Serum insulin concentrations were determined via immunoassay (ADVIA Centaur Autoanalyzer, Siemens Healthcare Diagnostics). The homeostasis model assessment of insulin resistance was estimated as follows: fasting insulin (µIU/mL) × fasting glucose (mmol/L)/22.5. Low-density lipoprotein cholesterol (LDL-c) was estimated using the Friedewald Eq. (19) [19]. Additional clinical data, such as diabetes duration and use of glucose-lowering therapies, were systematically recorded.

## Statistical 

### Statistical Analysis of Biochemical and Anthropometric Variables

Continuous variables are presented as mean ± standard deviation, and categorical variables as absolute numbers and percentages. Differences between groups were analyzed with the Mann-Whitney test for continuous variables, or Chi-square test for categorial variables. Values were considered to be statistically significant when *p* < 0.05.

## Gut Microbiota Analysis

### DNA Extraction and 16 S rRNA Sequencing

Microbial DNA was isolated from fecal samples utilizing the QIAamp DNA Stool Mini Kit (QIAGEN, Hilden, Germany) in accordance with the manufacturer’s guidelines.

Seven hypervariable regions (V2, V3, V4, V6, V7, V8, and V9) of the 16 S rRNA gene were amplified using the Ion 16 S™ Metagenomics Kit (Thermo Fisher Scientific, Waltham, MA, USA). The kit is designed to generate short amplicons spanning approximately 200–400 bp depending on the targeted region. Libraries were prepared using the Ion Plus Fragment Library Kit and Ion Xpress Barcodes Adapters, following the manufacturer’s protocol. (Thermo Fisher Scientific, Waltham, MA, USA). Template preparation and sequencing of the amplicon libraries were performed using the Ion 510/520/530TM Kit-Chef and the Ion Torrent S5TM system and (Thermo Fisher Scientific, Waltham, MA, USA) according to the manufacturer’s instructions.

### Data Processing

Sequencing data were processed using the Torrent Suite™ Software (version 5.18.1, ThermoFisher Scientific) for base calling and demultiplexing. Low-quality bases were trimmed at a minimum Phred score of 15, and reads were assigned to samples based on unique barcodes. Sequence failing quality thresholds or barcode assignment were removed prior to downstream processing.

Amplicon sequence variants (ASVs) were resolved using the DADA2 algorithm with adapted parameters for Ion Torrent data, within the open-source Quantitative Insights into Microbial Ecology software (QIIME2, version 2023.5). Features with fewer than 10 total reads across the dataset were removed. A prevalence filter was then applied, retaining only features present in at least two samples. Subsequently, sequences annotated as mitochondria or chloroplast were excluded to remove host- or plant-derived contaminants. Taxonomic assignment of ASVs was accomplished with the SILVA 138 database at 99% similarity, using reference files formatted for QIIME2 and processed with the RESCRIPt plugin.

### Diversity Analysis and Differential Abundance Analysis

Diversity assessments were performed in QIIME2 with the core-metrics-phylogenetic plugin following rarefaction to standardize sequencing depth. Alpha diversity (Observed Features, Faith’s Phylogenetic Diversity, Shannon index, Pielou’s Evenness) was analyzed via ANOVA. Beta diversity (Unweighted/Weighted UniFrac distances) was examined using PERMANOVA (Adonis test). Results were adjusted for potential pre-surgery confounders, such as age, HbA1c levels, T2D duration, and insulin treatment.

Both linear discriminant analysis effect size (LEfSe) and Analysis of Compositions of Microbiomes with Bias Correction 2 (ANCOM-BC2) were used to identify differentially abundant taxa between groups using Microbiomemarker R package (version 1.4.0) and ANCOMBC R package (version 2.0.3) in R studio (version 2023.09.1). In LEfSe analysis, non-parametric factorial Kruskal-Wallis sum-rank test was used to detect features with significant differential abundance (*p* < 0.05). Linear Discriminant Analysis (LDA) was performed to estimate the effect size of each differentially abundant feature. Taxa were considered significantly differentially abundant if *p* < 0.05 and LDA ≥ 2, whilst the ANCOM-BC2 model was adjusted for potential confounding factors, including age, duration of T2D, insulin treatment and HbA1c levels at baseline. Structural zeros were not further analysis using ANCOM-BC2. Results were considered significant at a p-value < 0.05.

Regression models were performed to study the potential role of gut microbiota in predicting T2D remission, including clinical covariates that significantly differed between groups and taxa identified as significantly differentially abundant by ANCOM-BC2. Count microbiota data were transformed using a centered log-ratio transformation after adding a pseudocounts to address compositional effects. The dataset was randomly split into a training set (70%) and an independent test set (30%), preserving class balance. Three predictive models were trained: (i) a clinical model using logistic regression (glm), (ii) a microbiota-only model including taxa identified as significant by ANCOM.BC2, trained using elastic net regression (glmnet), and (iii) a combined model integrating clinical and ANCOM-BC2-selected microbial features using glmnet. Model training and hyperparameter tuning were performed using repeated 5-fold cross-validation (20 repetitions), optimizing the area under the receiver operating characteristic (AUC) curve. Feature importance was extracted from the combined elastic net model.

### Functional Prediction

Functional profiles of the gut microbiota were predicted by Phylogenetic Investigation of Communities by Reconstruction of Unobserved States (PICRUSt2) plugin in QIIME2. The predicted functions output was analyzed in R studio using Limma voom in ggpicrust2 package. Pathways with p value < 0.05 were considered statistically significant.

## Results

### Anthropometric and Biochemical Characteristics

Forty-six participants were included in this study. The baseline characteristics of the study population are shown in Table 1. Prior to surgery, age, glucose levels, triglycerides levels, and HbA1c were significantly higher in non-remission group compared to remission group (Table 1). Also, a longer diabetes duration and higher percentage of baseline treatment with insulin therapy (considering patients on any insulin therapy preoperatively, encompassing both insulin-only and insulin plus other antidiabetic agents different from insulin) were observed in the non-remission group. The percentage of use of other glucose-lowering therapies different from insulin was not statistically significant between groups. As expected, both groups improved their metabolic status 1 year after SG, and only HbA1c remained significantly higher in the non-remission compared to the remission group (Table 1).

**Table 1:** Anthropometric and biochemical characteristics of patients included in the study

		Non-remission	Remission
Sex (M/F)		7/15	7/17
Age (years)		52.77 ± 8.00	49.25 ± 5.86*
Weight (kg)	Baseline	136.14 ± 18.52	129.70 ± 25.26
	1-year follow-up	101.21 ± 16.41	96.08 ± 19.83
BMI (kg/m2)	Baseline	49.98 ± 7.65	47.23 ± 6.54
	1-year follow-up	37.42 ± 7.75	35.07 ± 6.51
Glucose (mg/dl)	Baseline	144.36 ± 34.04	126.96 ± 36.44*
	1-year follow-up	112.38 ± 37.93	94.10 ± 12.48
Insulin (µUI/ml)	Baseline	31.04 ± 48.84	22.53 ± 17.46
	1-year follow-up	9.62 ± 6.23	11.31 ± 7.09
HOMA-IR	Baseline	9.95 ± 11.92	7.35 ± 6.47
	1-year follow-up	2.54 ± 1.80	2.72 ± 1.92
HbA1c (%)	Baseline	7.54 ± 1.41	6.79 ± 1.40*
	1-year follow-up	6.41 ± 1.33	5.65 ± 0.39^#^
Diabetes duration (years)		8.38 ± 3.98	4.40 ± 2.52*
Glucose-lowering medications	Baseline		
Metformin (n,%)		19 (86.4%)	22 (91.7%)
SGLT2i (n,%)		5 (22.7%)	6 (25.0%)
GLP-1 RA (n,%)		13 (59.1%)	9 (37.5%)
Insulin (n,%)		10 (45.5%)	1 (4.2%)*
Cholesterol (mg/dl)	Baseline	193.05 ± 37.69	179.42 ± 32.71
	1-year follow-up	185.86 ± 49.63	203.15 ± 31.36
Triglycerides (mg/dl)	Baseline	219.14 ± 99.87	164.71 ± 82.20*
	1-year follow-up	132.90 ± 64.82	107.00 ± 34.79
HDL cholesterol (mg/dl)	Baseline	42.32 ± 8.11	42.75 ± 10.11
	1-year follow-up	49.38 ± 10.14	52.90 ± 12.63
LDL cholesterol (mg/dl)	Baseline	111.56 ± 32.35	103.75 ± 26.67
	1-year follow-up	118.08 ± 47.22	128.83 ± 24.11

*p < 0.05. Mann-Whitney/Chi-square test for comparison between non-remission and remission group at baseline. # *p* < 0.05. Mann-Whitney test for comparison between non-remission and remission group at 1-year follow-up. HDL, high-density lipoprotein; LDL, low-density lipoprotein, SGLT2i, sodium-glucose cotransporter 2 inhibitors, GLP-1 RA, glucagon-like peptide 1 receptor agonists

### Gut Microbiota Diversity

**Fig. 1:**
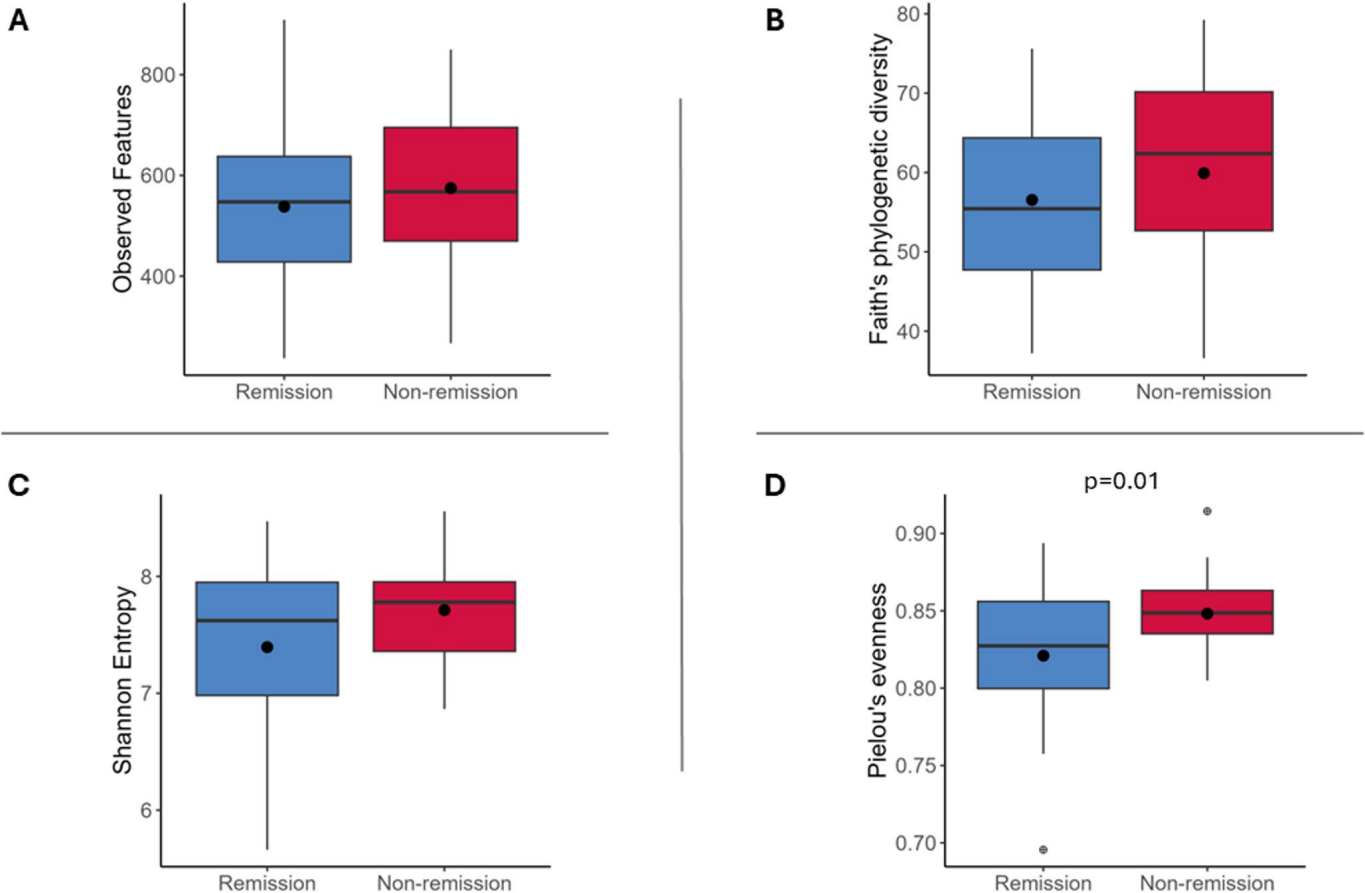
Gut microbiota diversity. **A**. Observed features. **B **Faith’s phylodiversity. **C**. Shannon. **D**. Pielou’s evenness

After adjusting for potential baseline confounders, including age, HbA1c, duration of T2D and insulin treatment, the alpha-diversity analysis showed an increase in Pielou’s evenness index in non-remission group compared to remission group (*p* = 0.01), Fig. 1. No statistically significant differences were found in the rest of alpha-diversity and beta-diversity indexes.

### Gut Microbiota Composition According to T2D Remission After Sleeve Gastrectomy

**Fig. 2:**
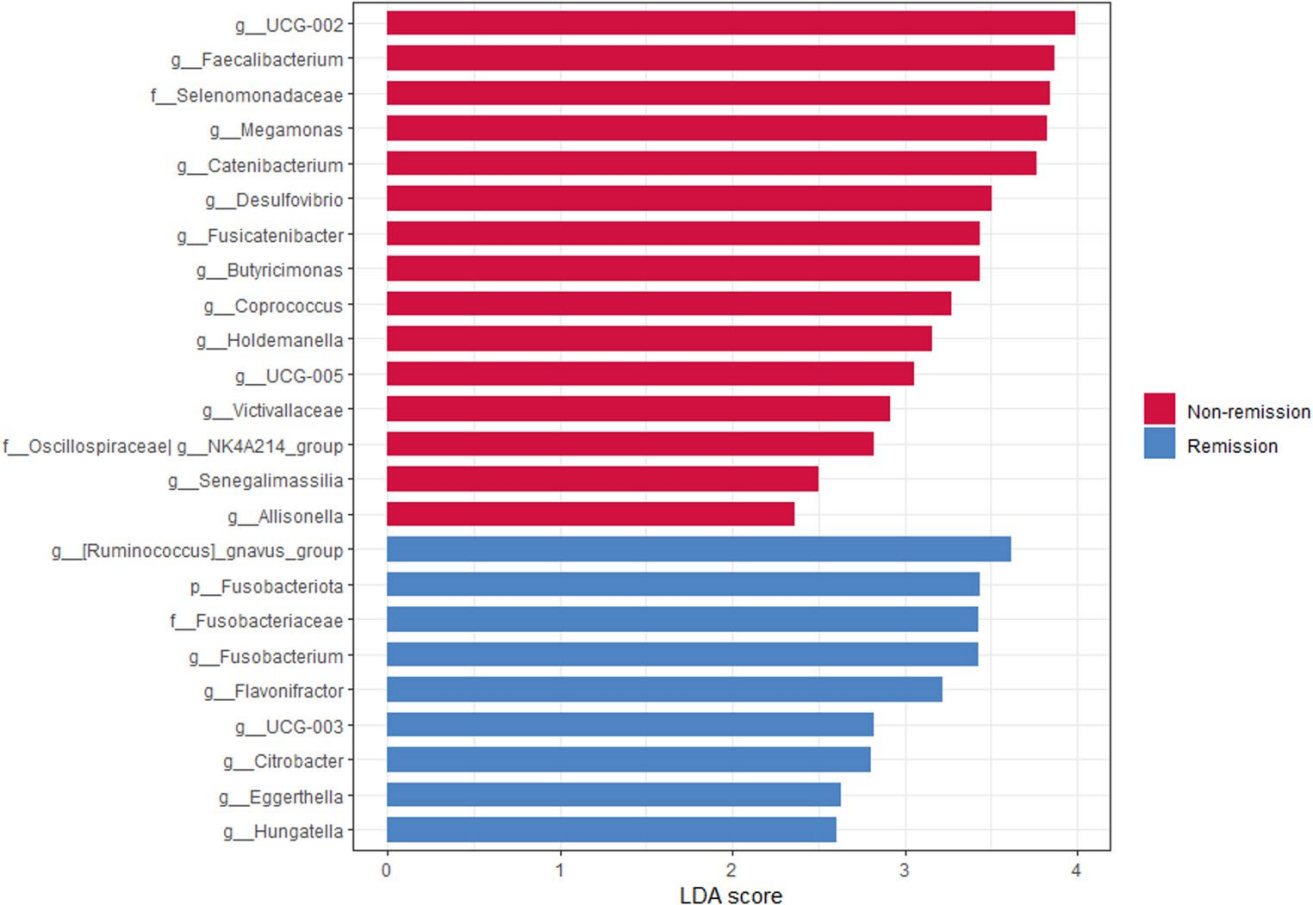
Significantly different taxa identified by linear discriminant analysis effect size (LEFSe) according to T2D remission (LDA score > 2; *p* < 0.05)

The initial DADA2 feature table contained 5,831,727 total sequences across 12,199 ASVs. After applying abundance and prevalence filters, the final dataset comprised 5,166,726 sequences (88.6% retained) and 4,558 ASVs. The analysis of differences in gut microbiota composition at baseline by LefSe revealed that the non-remission group was enriched in *Faecalibacterium*, *Allisonella*, *Megamonas*, *Desulfovibrio*, *Butyricimonas*, *Victivallaceae*, *Senegalimassilia* and members of *Oscillospiracea* family (*UCG-002*, *UCG-005* and *NK4A214*), *Lachnospiraceae* family (*Fusicatenibacter* and *Coprococcus*), and *Erysipelatoclostridiaceae* family (*Catenibacterium* and *Holdemanella*), whilst the remission group was shown to be enriched in *Eggerthella*, *Fusobacterium*, *Citrobacter*, and members of *Oscillospiraceae* family (*Flavonifractor* and *UCG-003*), and *Lachnospiraceae* family (*Ruminococcus gnavus group* and *Hungatella*) (Fig. 2).

**Fig. 3:**
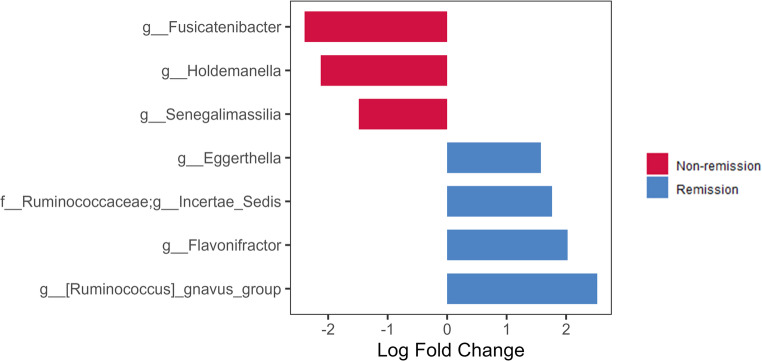
Differentially abundant taxa identified by ANCOM-BC2 adjusted by age, HbA1c levels, duration of T2D and insulin treatment prior to surgery

After adjusting for age, baseline HbA1c levels, duration of T2D, and insulin treatment, the ANCOM-BC2 analysis revealed an enrichment in *Fusicatenibacter*, *Holdemanella* and *Senegalimassilia* in the non-remission group, whilst *Eggerthella*,* Flavonifractor*,* Ruminococcaceae g__Incertae Sedis* and *R. gnavus group* were enriched in remission group (Fig. 3).

To further explore the potential role of the gut microbiota as maker for T2D remission, cross-validating regression models were performed. The clinical model, including insulin treatment, diabetes duration, HbA1c, BMI, triglycerides, and age, showed modest discriminatory ability (AUC = 0.660). The microbiota-only model, based on taxa identified as significant by ANCOM-BC2, achieved higher predictive performance (AUC = 0.800). The combined model, integrating both clinical variables and ANCOM-BC2–selected microbial taxa, showed the highest predictive performance (AUC = 0.928), with balanced sensitivity (0.818) and specificity (0.872) (Supplementary Fig. 2A).

The combined model suggests that insulin treatment was the strongest single clinical predictor, accounting for approximately 27% of total model importance, followed by disease duration. Regarding to gut microbiota, *Ruminococcaceae g__Incertae Sedis*, *Fusicatenibacter* and *Eggerthella* were the taxa with the highest predictive contributions (Supplementary Fig. 2B).

### Predictive Differential Abundance of Metabolic Pathways

**Fig. 4:**
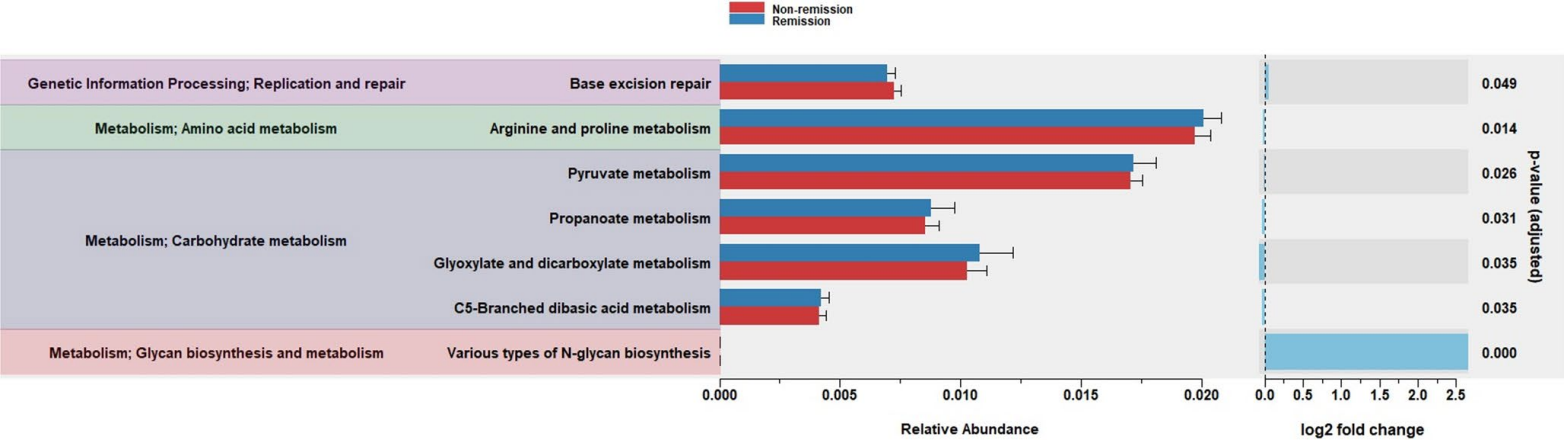
Predictive differential abundance of metabolic pathways by PICRUSt2

Predicted functional analysis based on PICRUSt2 is shown in Fig. 4. We found that pathways involved in carbohydrate metabolism, such as pyruvate metabolism, propanoate metabolism, glyoxylate and dicarboxylate metabolism and C5-branched dibasic acid metabolism were enriched in the remission group.

## Discussion

In this study, we show that distinct gut microbiota composition patterns can be observed preoperatively in patients who achieve remission of T2D following SG compared to those who do not. Therefore, our results suggest that the gut microbiome might become a potential biomarker for predicting T2D remission after SG.

Previous evidence supports that the gut microbiome may serve as a predictor of BS-related outcomes, such as weight loss. In line with this, it was previously found that the Prevotella-to-Bacteroides ratio could be a useful predictive tool for weight loss trajectories after SG [11]. However, little is known about the role of the preoperative gut microbiota in the improvement or resolution of different metabolic comorbidities associated with obesity. To our knowledge, the study by Davies et al. was the main report to date evaluating the potential role of baseline gut microbiome as a predictor of T2D resolution after BS [16]. However, this study included only a small sample of patients undergoing SG (*n* = 22) and did not analyze the study population separately (i.e., patients who underwent RYGB or SG) to assess this outcome. Therefore, given that different mechanisms in the remission of T2D following SG or RYGB have been postulated [20], distinct gut microbiota predictive patterns might also be expected. Additionally, this study did not adjust for baseline confounders known to be associated with T2D remission after BS. On the other hand, results from the CORDIOPREV trial showed that a specific gut microbiota profile may be associated with diet-induced T2D remission [21], suggesting, therefore, that the gut microbiome could potentially serve as predictor of the response to different therapeutic approaches in the management of T2D.

Notably, we did not observe significant differences in bacterial richness or diversity between the remission and non-remission groups, except for Pielou’s evenness. These results are in line with our previous findings regarding the predictive role of gut microbiota in weight loss following BS [11], and may suggest that pre-surgery gut microbiota composition, rather than diversity, plays a predominant role in T2D remission, as similarly reported in some lifestyle interventions [21].

We observed differences in preoperative gut microbiota composition in individuals with and without T2D remission following SG. On the one hand, an enrichment of *R. gnavus* at baseline was detected in the remission group of our study. This bacterium has been associated with metabolic diseases, including metabolic dysfunction-associated steatotic liver disease [22]. Cross-sectional associations between *R. gnavus* and features of the metabolic syndrome have also been described [23], and previous data suggest a role in the development of some complications related to T2D [24]. Importantly, an enrichment in the genera *Eggerthella* and *Flavonifractor* was also found after adjusting for potential confounders, and these were also the main genera able to predict T2D remission after SG. The genus *Eggerthella*, and some of its species, such as *Eggerthella lenta*, have been associated with T2D [25, 26] and a reduced abundance may be found in subjects with a healthy metabolic profile [27]. Additionally, this genus has been associated with the progression from prediabetes to T2D [28], and with certain T2D-related microvascular complications, such as diabetic neuropathy [29, 30]. It should also be noted that an increased abundance of *Eggethella* may be associated with alterations in gut microbiota composition and function in some inflammatory diseases, such as inflammatory bowel disease or rheumatoid arthritis [31, 32]. Conversely, lower abundances of *Eggerthella lenta* have been reported following SG or RYGB compared with pre-surgery levels, suggesting a relationship between metabolic improvement and changes in this bacterium [33]. Interestingly, despite being a butyrate-producing genus, *Flavonifractor* has been associated with reduced insulin sensitivity, suggesting a direct link to an adverse metabolic profile [34]. These results align with previous reports [16], which, similar to ours, observed a higher abundance of potential biomarkers of altered gut microbiota composition at baseline, such as *Alistipes putredinis*, in subjects with T2D remission after BS. On the other hand, an increase in the abundance of certain bacteria linked to improvements in the metabolic profile, such as *Fusicatenibacter, Senegalimassilia* and *Holdemanella* [35, 36], was identified in the non-remission group. In a diet-induced obese mouse model, *Holdemanella*, has been shown to ameliorate hyperglycaemia, contributing to improve glucose tolerance [36]. Furthermore, *Fusicatenibacter* has been identified as a key short chain fatty acid producer which play a crucial role in gut health [37] and has been shown to be decreased in patients with T2D [35]. On the other hand, *Senegalimassilia* has been shown to be a protective factor of metabolic diseases such as hypertension and metabolic syndrome [38, 39].

Taking all these together, our findings may suggest that a baseline gut microbiota profile associated with insulin resistance might ultimately result in an enhanced response to SG in terms of T2D remission.

Finally, we conducted an exploratory functional analysis to assess potential differences in gut microbiota-associated pathways between groups. Interestingly, we observed an enrichment in pathways related to carbohydrate metabolism in the remission group. Previous studies have reported alterations in dicarboxylate metabolism in T2D and other metabolic diseases [40], and impairments in branched-chain amino acid metabolism may also be found in T2D [41]. Thus, these findings might indicate an enhanced response to SG in patients with a specific gut microbial functional profile, although further research is warranted.

This study has several limitations. Hence, although this this is the largest study to date exclusively including SG, our results should be cautiously interpreted, and considered as preliminary, due to the small sample size. Additionally, T2D remission was assessed only in the short term; thus, further long-term studies are needed to evaluate the impact of baseline gut microbiota composition on this outcome. Information regarding physical activity or diet at baseline was not specifically recorded. Also, the functional analyses conducted using PICRUSt2 should be regarded as hypothesis-generating. On the other hand, some important strengths should be highlighted. First, participants were closely followed up throughout the study period. Furthermore, considering that various preoperative factors have been reported significatively influence T2D remission following BS and may also affect baseline gut microbiota composition, a careful adjustment for potential confounders was incorporated into analyses.

## Conclusions

In patients with T2D undergoing SG, baseline gut microbiota composition may serve as an independent predictor of diabetes remission. Also, our results suggest that gut microbiota could be a potentially relevant biomarker of diabetes remission, complementing other existing clinical predictors. However, further research is required to confirm these findings and explore their long-term implications.

## Supplementary Information

Below is the link to the electronic supplementary material.

Additional file 1. Supplementary Figure 1: Flowchart of the study participants.

Additional file 2. Supplementary Figure 2: A. Receiver operating characteristic curves. Clinical model: including age, body mass index, triglyceride levels, insulin treatment, diabetes duration, and HbA1c. Microbiota model including significantly different taxa identified by ANCOM-BC2. Combined model including both clinical variables and taxa. B. Feature importance normalized to percentage of total contribution in combined model.

## Data Availability

All data used for the analysis in this article are available on request from the corresponding authors. The 16 S raw sequencing data generated in this study are available through the NCBI Sequence Read Archive under project number PRJNA1235123.

## References

[1] Saeedi P, Petersohn I, Salpea P, Malanda B, Karuranga S, Unwin N, et al. Global and regional diabetes prevalence estimates for 2019 and projections for 2030 and 2045: Results from the International Diabetes Federation Diabetes Atlas, 9th edition. Diabetes Res Clin Pract. 2019;157:107843. 10.1016/j.diabres.2019.107843. 31518657 10.1016/j.diabres.2019.107843

[2] Ahmad E, Lim S, Lamptey R, Webb DR, Davies MJ. Type 2 diabetes. Lancet. 2022;400(10365):1803–20. 10.1016/S0140-6736(22)01655-5. 36332637 10.1016/S0140-6736(22)01655-5

[3] Yang G, Wei J, Liu P, Zhang Q, Tian Y, Hou G, et al. Role of the gut microbiota in type 2 diabetes and related diseases. Metabolism. 2021;117:154712. 10.1016/j.metabol.2021.154712. 33497712 10.1016/j.metabol.2021.154712

[4] Byndloss M, Devkota S, Duca F, Hendrik Niess J, Nieuwdorp M, Orho-Melander M, et al. The Gut Microbiota and Diabetes: Research, Translation, and Clinical Applications—2023 Diabetes, Diabetes Care, and Diabetologia Expert Forum. Diabetes Care. 2024. 10.2337/dci24-0052. 38996003 10.2337/dci24-0052 PMC11362125

[5] Mingrone G, Panunzi S, De Gaetano A, Guidone C, Iaconelli A, Capristo E, et al. Metabolic surgery versus conventional medical therapy in patients with type 2 diabetes: 10-year follow-up of an open-label, single-centre, randomised controlled trial. Lancet. 2021;397(10271):293–304. 10.1016/S0140-6736(20)32649-0. 33485454 10.1016/S0140-6736(20)32649-0

[6] Courcoulas AP, Patti ME, Hu B, Arterburn DE, Simonson DC, Gourash WF, et al. Long-Term Outcomes of Medical Management vs Bariatric Surgery in Type 2 Diabetes. JAMA. 2024;331(8):654. 10.1001/jama.2024.0318. 38411644 10.1001/jama.2024.0318 PMC10900968

[7] Hernández-Montoliu L, Rodríguez-Peña M-M, Puig R, Astiarraga B, Guerrero-Pérez F, Virgili N, et al. A specific gut microbiota signature is associated with an enhanced GLP-1 and GLP-2 secretion and improved metabolic control in patients with type 2 diabetes after metabolic Roux-en-Y gastric bypass. Front Endocrinol (Lausanne). 2023;14. 10.3389/fendo.2023.1181744. 10.3389/fendo.2023.1181744 PMC10616869 37916149

[8] Debédat J, Le Roy T, Voland L, Belda E, Alili R, Adriouch S, et al. The human gut microbiota contributes to type-2 diabetes non-resolution 5-years after Roux-en-Y gastric bypass. Gut Microbes. 2022;14(1). 10.1080/19490976.2022.2050635. 10.1080/19490976.2022.2050635 PMC9037437 35435140

[9] Murphy R, Tsai P, Jüllig M, Liu A, Plank L, Booth M. Differential Changes in Gut Microbiota After Gastric Bypass and Sleeve Gastrectomy Bariatric Surgery Vary According to Diabetes Remission. Obes Surg. 2017;27(4):917–25. 10.1007/s11695-016-2399-2. 27738970 10.1007/s11695-016-2399-2

[10] Wang R, Mijiti S, Xu Q, Liu Y, Deng C, Huang J, et al. The Potential Mechanism of Remission in Type 2 Diabetes Mellitus After Vertical Sleeve Gastrectomy. Obes Surg. 2024;34(8):3071–83. 10.1007/s11695-024-07378-z. 38951388 10.1007/s11695-024-07378-z

[11] Gutiérrez-Repiso C, Garrido-Sánchez L, Alcaide-Torres J, Cornejo-Pareja I, Ocaña-Wilhelmi L, García-Fuentes E, et al. Predictive Role of Gut Microbiota in Weight Loss Achievement after Bariatric Surgery. J Am Coll Surg. 2022;234(5):861–71. 10.1097/XCS.0000000000000145. 35426398 10.1097/XCS.0000000000000145

[12] Fouladi F, Carroll IM, Sharpton TJ, Bulik-Sullivan E, Heinberg L, Steffen KJ, et al. A microbial signature following bariatric surgery is robustly consistent across multiple cohorts. Gut Microbes. 2021;13(1). 10.1080/19490976.2021.1930872. 10.1080/19490976.2021.1930872 PMC8224199 34159880

[13] Panunzi S, De Gaetano A, Carnicelli A, Mingrone G. Predictors of remission of diabetes mellitus in severely obese individuals undergoing bariatric surgery: do BMI or procedure choice matter? A meta-analysis. Ann Surg. 2015;261(3):459–67. 10.1097/SLA.0000000000000863. 25361217 10.1097/SLA.0000000000000863

[14] Blackstone R, Bunt JC, Cortés MC, Sugerman HJ. Type 2 diabetes after gastric bypass: remission in five models using HbA1c, fasting blood glucose, and medication status. Surg Obes Relat Dis. 2012;8(5):548–55. 10.1016/j.soard.2012.05.005. 22721581 10.1016/j.soard.2012.05.005

[15] Still CD, Wood GC, Benotti P, Petrick AT, Gabrielsen J, Strodel WE, et al. Preoperative prediction of type 2 diabetes remission after Roux-en-Y gastric bypass surgery: a retrospective cohort study. Lancet Diabetes Endocrinol. 2014;2(1):38–45. 10.1016/S2213-8587(13)70070-6. 24579062 10.1016/S2213-8587(13)70070-6 PMC3932625

[16] Davies N, O’Sullivan JM, Plank LD, Murphy R. Gut Microbial Predictors of Type 2 Diabetes Remission Following Bariatric Surgery. Obes Surg. 2020;30(9):3536–48. 10.1007/s11695-020-04684-0. 32447634 10.1007/s11695-020-04684-0

[17] Al Assal K, Prifti E, Belda E, Sala P, Clément K, Dao M-C, et al. Gut Microbiota Profile of Obese Diabetic Women Submitted to Roux-en-Y Gastric Bypass and Its Association with Food Intake and Postoperative Diabetes Remission. Nutrients. 2020;12(2):278. 10.3390/nu12020278. 31973130 10.3390/nu12020278 PMC7071117

[18] Riddle MC, Cefalu WT, Evans PH, Gerstein HC, Nauck MA, Oh WK, et al. Consensus Report: Definition and Interpretation of Remission in Type 2 Diabetes. Diabetes Care. 2021;44(10):2438–44. 10.2337/dci21-0034. 34462270 10.2337/dci21-0034 PMC8929179

[19] Friedewald WT, Levy RI, Fredrickson DS. Estimation of the concentration of low-density lipoprotein cholesterol in plasma, without use of the preparative ultracentrifuge. Clin Chem. 1972;18(6):499–502. 4337382

[20] Nannipieri M, Baldi S, Mari A, Colligiani D, Guarino D, Camastra S, et al. Roux-en-Y Gastric Bypass and Sleeve Gastrectomy: Mechanisms of Diabetes Remission and Role of Gut Hormones. J Clin Endocrinol Metab. 2013;98(11):4391–9. 10.1210/jc.2013-2538. 24057293 10.1210/jc.2013-2538

[21] Vals-Delgado C, Alcala‐Diaz JF, Roncero‐Ramos I, Leon‐Acuña A, Molina‐Abril H, Gutierrez‐Mariscal FM, et al. A microbiota‐based predictive model for type 2 diabetes remission induced by dietary intervention: From the CORDIOPREV study. Clin Transl Med. 2021;11(4). 10.1002/ctm2.326. 10.1002/ctm2.326 PMC8023646 33931973

[22] Meadows V, Antonio JM, Ferraris RP, Gao N. Ruminococcus gnavus in the gut: driver, contributor, or innocent bystander in steatotic liver disease? FEBS J. 2024. 10.1111/febs.17327. 39589934 10.1111/febs.17327 PMC11927045

[23] Grahnemo L, Nethander M, Coward E, Gabrielsen ME, Sree S, Billod J-M, et al. Cross-sectional associations between the gut microbe Ruminococcus gnavus and features of the metabolic syndrome: the HUNT study. Lancet Diabetes Endocrinol. 2022;10(7):481–3. 10.1016/S2213-8587(22)00113-9. 35662399 10.1016/S2213-8587(22)00113-9

[24] Hong J, Fu T, Liu W, Du Y, Bu J, Wei G, et al. Specific Alternation of Gut Microbiota and the Role of Ruminococcus gnavus in the Development of Diabetic Nephropathy. J Microbiol Biotechnol. 2024;34(3):547–61. 10.4014/jmb.2310.10028. 38346799 10.4014/jmb.2310.10028 PMC11016775

[25] Qin J, Li Y, Cai Z, Li S, Zhu J, Zhang F, et al. A metagenome-wide association study of gut microbiota in type 2 diabetes. Nature. 2012;490(7418):55–60. 10.1038/nature11450. 23023125 10.1038/nature11450

[26] Baars DP, Fondevila MF, Meijnikman AS, Nieuwdorp M. The central role of the gut microbiota in the pathophysiology and management of type 2 diabetes. Cell Host Microbe. 2024;32(8):1280–300. 10.1016/j.chom.2024.07.017. 39146799 10.1016/j.chom.2024.07.017

[27] Bakir-Gungor B, Bulut O, Jabeer A, Nalbantoglu OU, Yousef M. Discovering Potential Taxonomic Biomarkers of Type 2 Diabetes From Human Gut Microbiota via Different Feature Selection Methods. Front Microbiol. 2021;12. 10.3389/fmicb.2021.628426. 10.3389/fmicb.2021.628426 PMC8424122 34512559

[28] Zhang B, Zhang X, Luo Z, Ren J, Yu X, Zhao H, et al. Microbiome and metabolome dysbiosis analysis in impaired glucose tolerance for the prediction of progression to diabetes mellitus. J Genet Genomics. 2024;51(1):75–86. 10.1016/j.jgg.2023.08.005. 37652264 10.1016/j.jgg.2023.08.005

[29] Zhou P, Hao Z, Chen Y, Zhang Z, Xu W, Yu J. Association between gut microbiota and diabetic microvascular complications: a two-sample Mendelian randomization study. Front Endocrinol (Lausanne). 2024;15. 10.3389/fendo.2024.1364280. 10.3389/fendo.2024.1364280 PMC11327146 39157683

[30] Xie L, Gan W, Cai G. The causal relationship between gut microbiota and diabetic neuropathy: a bi-directional two-sample Mendelian randomization study. Front Endocrinol (Lausanne). 2024;15. 10.3389/fendo.2024.1402014. 10.3389/fendo.2024.1402014 PMC11266094 39050567

[31] Alexander M, Ang QY, Nayak RR, Bustion AE, Sandy M, Zhang B, et al. Human gut bacterial metabolism drives Th17 activation and colitis. Cell Host Microbe. 2022;30(1):17–e309. 10.1016/j.chom.2021.11.001. 34822777 10.1016/j.chom.2021.11.001 PMC8785648

[32] Balakrishnan B, Luckey D, Wright K, Davis JM, Chen J, Taneja V. Eggerthella lenta augments preclinical autoantibody production and metabolic shift mimicking senescence in arthritis. Sci Adv. 2023;9(35). 10.1126/sciadv.adg1129. 10.1126/sciadv.adg1129 PMC10854426 37656793

[33] Hussan H, Clinton SK, Grainger EM, Webb M, Wang C, Webb A, et al. Distinctive patterns of sulfide- and butyrate-metabolizing bacteria after bariatric surgery: potential implications for colorectal cancer risk. Gut Microbes. 2023;15(2). 10.1080/19490976.2023.2255345. 10.1080/19490976.2023.2255345 PMC10501170 37702461

[34] Cui J, Ramesh G, Wu M, Jensen ET, Crago O, Bertoni AG, et al. Butyrate-Producing Bacteria and Insulin Homeostasis: The Microbiome and Insulin Longitudinal Evaluation Study (MILES). Diabetes. 2022;71(11):2438–46. 10.2337/db22-0168. 35972231 10.2337/db22-0168 PMC9630078

[35] Siptroth J, Moskalenko O, Krumbiegel C, Ackermann J, Koch I, Pospisil H. Variation of butyrate production in the gut microbiome in type 2 diabetes patients. Int Microbiol. 2023;26(3):601–10. 10.1007/s10123-023-00324-6. 36780038 10.1007/s10123-023-00324-6 PMC10397123

[36] Romaní-Pérez M, López‐Almela I, Bullich‐Vilarrubias C, Rueda‐Ruzafa L, Del Gómez EM, Benítez‐Páez A, et al. Holdemanella biformis improves glucose tolerance and regulates GLP‐1 signaling in obese mice. FASEB J. 2021;35(7). 10.1096/fj.202100126R. 10.1096/fj.202100126R 34143451

[37] Gryaznova M, Smirnova Y, Burakova I, Syromyatnikov M, Chizhkov P, Popov E, et al. Changes in the Human Gut Microbiome Caused by the Short-Term Impact of Lactic Acid Bacteria Consumption in Healthy People. Probiotics Antimicrob Proteins. 2024;16(4):1240–50. 10.1007/s12602-023-10111-4. 37365419 10.1007/s12602-023-10111-4

[38] Li Y, Fu R, Li R, Zeng J, Liu T, Li X, et al. Causality of gut microbiome and hypertension: A bidirectional mendelian randomization study. Front Cardiovasc Med. 2023;10. 10.3389/fcvm.2023.1167346. 10.3389/fcvm.2023.1167346 PMC10192878 37215554

[39] Zhao H, Zhu G, Zhu T, Ding B, Xu A, Gao S, et al. Gut microbiome and metabolism alterations in schizophrenia with metabolic syndrome severity. BMC Psychiatry. 2024;24(1):529. 10.1186/s12888-024-05969-9. 39048972 10.1186/s12888-024-05969-9 PMC11267952

[40] Proffitt C, Bidkhori G, Lee S, Tebani A, Mardinoglu A, Uhlen M, et al. Genome-scale metabolic modelling of the human gut microbiome reveals changes in the glyoxylate and dicarboxylate metabolism in metabolic disorders. iScience. 2022;25(7):104513. 10.1016/j.isci.2022.104513. 35754734 10.1016/j.isci.2022.104513 PMC9213702

[41] Sjögren RJO, Rizo-Roca D, Chibalin AV, Chorell E, Furrer R, Katayama S, et al. Branched-chain amino acid metabolism is regulated by ERRα in primary human myotubes and is further impaired by glucose loading in type 2 diabetes. Diabetologia. 2021;64(9):2077–91. 10.1007/s00125-021-05481-9. 34131782 10.1007/s00125-021-05481-9 PMC8382616

