# Supplementary figures and images for: Gut Microbiota-specific Profile Prior to Surgery for Predicting Type 2 Diabetes Remission in Patients Undergoing Sleeve Gastrectomy
Source: Obes Surg. 2026 Mar 26;36(5):2270–80. doi: 10.1007/s11695-026-08612-6 (PMC13222206; doi:10.1007/s11695-026-08612-6)

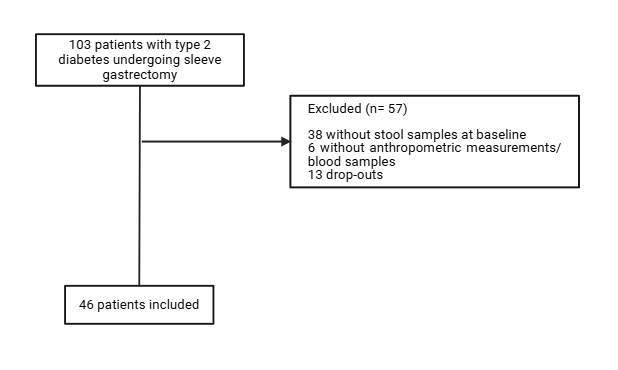

Supplement: Supplementary file 1 — Additional file 1. Supplementary Figure 1: Flowchart of the study participants. [file 11695_2026_8612_MOESM1_ESM.png]

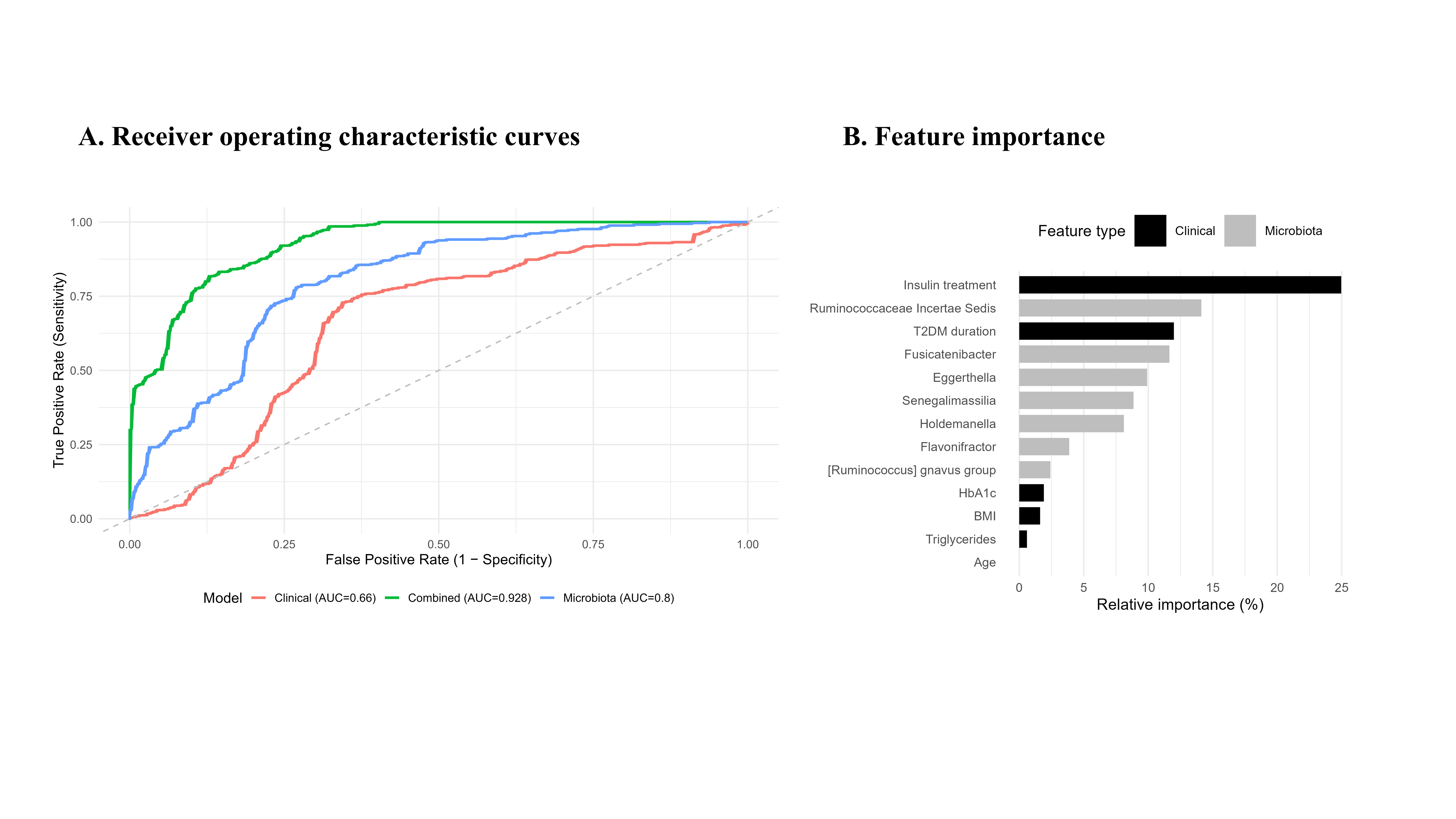

Supplement: Supplementary file 2 — Additional file 2. Supplementary Figure 2: A. Receiver operating characteristic curves. Clinical model: including age, body mass index, triglyceride levels, insulin treatment, diabetes duration, and HbA1c. Microbiota model including significantly different taxa identified by ANCOM-BC2. Combined model including both clinical variables and taxa. B. Feature importance normalized to percentage of total contribution in combined model. [file 11695_2026_8612_MOESM2_ESM.tif]
